# MicroRNA‐374b mediates the initiation of non‐small cell lung cancer by regulating ITGB1 and p53 expressions

**DOI:** 10.1111/1759-7714.13457

**Published:** 2020-05-04

**Authors:** Meng Zhao, Chuntang Tong, Zerui Hao, Ruixing Zhao, Liming Wang

**Affiliations:** ^1^ Department of Respiratory Medicine The Second People's Hospital of Liaocheng Linqing China; ^2^ Department of Thoracic Surgery The Second People's Hospital of Liaocheng Linqing China; ^3^ Department of Respiratory Medicine Weifang People's Hospital Weifang China

**Keywords:** ITGB1, miR‐374b, non‐small cell lung cancer, p53

## Abstract

**Background:**

Previous studies have shown that microRNAs (miRNAs) play important roles in the pathogenesis of human cancers. This study aims to clarify the role of miR‐374b in non‐small cell lung cancer (NSCLC).

**Methods:**

In this study, RT‐qPCR and western blot analysis were used to measure mRNA and protein expression. The regulatory mechanism of miR‐374b/ITGB1 was investigated by dual‐luciferase reporter, CCK‐8, and transwell assays.

**Results:**

MiR‐374b expression was reduced in NSCLC tissues and associated with lymph node metastasis, tumor stage and prognosis in NSCLC patients. Functionally, overexpression of miR‐374b inhibited cell viability and metastasis in NSCLC. In addition, miR‐374b blocked EMT and promoted p53 expression in NSCLC. MiR‐374b was found to directly target ITGB1. Furthermore, upregulation of ITGB1 weakened the antitumor effect of miR‐374b in NSCLC.

**Conclusions:**

MiR‐374b inhibits the tumorigenesis of NSCLC by downregulating ITGB1 and upregulating p53.

## Introduction

Non‐small cell lung cancer (NSCLC) is a malignant tumor with cancer cells originating in the lung.^1^ Each type of NSCLC is composed of different cancer cells, and their growth and spread are also inconsistent. Compared with small cell carcinoma, NSCLC cells grow and divide more slowly. Moreover, the proliferation and metastasis of NSCLC cells are relatively late.^2^ However, approximately 75% of NSCLC patients are already in advanced stages when they are diagnosed and the prognosis is worse.^3^ Therefore, the early diagnosis of NSCLC patients is of great significance.

It has been recognized that microRNAs (miRNAs) regulate target genes by splicing the transcription products of target genes or inhibiting the translation of transcription products.^4^ Mature miRNAs are evolutionarily conserved and can regulate the expression of related genes in human diseases. Researchers speculate that one‐third of human genes are regulated by miRNAs.^5^ Meanwhile, many miRNAs have been investigated and show different effects in NSCLC. For example, miR‐577 inhibits cell proliferation and epithelial‐mesenchymal transition (EMT) in NSCLC.^6^ Conversely, miR‐21 promotes cell proliferation, migration and invasion in NSCLC.^7^ Now, due to the dysregulation of miR‐374b function in human cancer, it has attracted our attention. For example, miR‐374b expression has been found to be reduced in pancreatic cancer, thereby promoting chemotherapeutic resistance.^8^ However, increased expression of miR‐374b has been detected in gastrointestinal stromal tumors and promoted cell proliferation.^9^ In addition, p53/miR‐374b has been shown to regulate the development of colorectal cancer.^10^ Sun *et al*. proposed that miR‐138 regulated p53 expression to inhibit NSCLC progression.^11^ The dysregulation of tumor suppressor gene p53 has been discovered in the origin of human cancer.^12^ Therefore, the effect of miR‐374b on p53 was investigated in NSCLC.

The abnormal expression of integrin beta 1 (ITGB1) has been found in several malignant tumors, such as breast, prostate cancer and pancreatic cancer.^13^ Functionally, it has been found that ITGB1 expression can regulate cell‐matrix adhesion and alter with breast cancer progression.^14^ In addition, downregulation of ITGB1 was found to inhibit the progression of esophageal squamous cell carcinoma.^15^ In particular, it has been reported that high expression of miR‐493‐5p predicted the clinical prognosis of NSCLC patients by targeting the oncogene ITGB1.^16^ Although ITGB1 has been reported to be the target gene of several miRNAs, the relationship between miR‐374b and ITGB1 has not been previously reported.

This study investigated the expression of miR‐374b in NSCLC and its relationship with ITGB1. The function of miR‐374b on tumorigenesis of NSCLC was detected in NSCLC cells and future clinical applications may require further research.

## Methods

### Experimental sample

NSCLC tissues and paracancerous normal tissues were obtained from The Second People's Hospital of Liaocheng. The patients with NSCLC who provided informed consent received only surgery. This study was approved by the Institutional Ethics Committee of The Second People's Hospital of Liaocheng.

### Cell culture and transfection

Human bronchial epithelial cells (16HBE) and H1299 NSCLC cell lines were obtained from ATCC (Manassas, VA, USA). The culture conditions of these cells were RPMI‐1640 medium, 10% FBS, 5% CO_2_, and 37°C. MiR‐374b mimics or inhibitor and ITGB1 vector were purchased from Genechem (Shanghai, China). They were then transfected into H1299 cells using Lipofectamine 2000. Untreated H1299 cells were used as a negative control (NC).

### RT‐qPCR

The extraction of mRNA was performed using TRIzol reagent (Invitrogen, Carlsbad, USA). RT‐qPCR was performed using SYBR Green Master Mix II (Takara) and corresponding primers. U6 or GAPDH was standardized as endogenous controls by miR‐374b or ITGB1. The relative expression of miR‐374b or ITGB1 was detected by 2^−△△ct^ method. The primers are shown in Table 1.

**Table 1:** The sequence of RT‐qPCR primers

Primers	Sequence
miR‐374b	F: 5'‐AUA UAA UAC AAC CUG CUA AGU G‐3'
	R: 5'‐TTC ACG AAT TTG CGT GTC AT‐3
U6	F: 5'‐CTC GCT TCG GCA GCA CA‐3'
	R: 5'‐AAC GCT TCA CGA ATT TGC GT‐3'
ITGB1	F: 5′‐AAT GTA ACC AAC CGT AGC‐3'
	R: 5'‐CAG GTC CAT AAG GTA GTA GA‐3'
GAPDH	F, 5'‐ACA TCG CTC AGA CAC CAT G‐3'
	5'‐TGT AGT TGA GGT CAA TGA AGG G‐3'

### CCK‐8 assay

CK‐8 (Dojindo, Kumamoto, Japan) solution was purchased to evaluate cell proliferation according to product instructions. The experimental procedure was performed based on a previous study.^17^

### Transwell assay

Cell migration and invasion were detected by transwell chambers with or without Matrigel. H1299 cells (5 × 10^3^ cells/well) were added to the upper chamber. The lower chamber was filled with RPMI‐1640 medium containing 10% FBS. Migrated or invasive cells were stained with 0.1% crystal violet. An Olympus microscope was used to count the migrated and invaded cells.

### Luciferase reporter assay

The pcDNA3.1 plasmid vector (Promega, Madison, USA) was inserted into the 3'‐UTR of wild‐type or mutant ITGB1. The plasmid and miR‐374b mimics were transfected into H1299 cells. Finally, we measured the luciferase activity using a dual luciferase assay system (Promega, USA).

### Western blot analysis

RIPA buffer (Applygen, Beijing, China) was used for protein lyses. The protein samples were separated by 10% SDS‐PAGE protein and transferred into PVDF membrane. After blocking with 5% skim milk, the membrane was incubated with E‐cadherin, N‐cadherin, vimentin, ITGB1, p53 and GAPDH primary antibodies overnight at 4°C. After that, the corresponding secondary antibody was added, and the membrane was incubated for two hours. Protein bands were observed by ECL reagent (Millipore, MA, USA).

### Statistical analysis

Data were analyzed using SPSS 18.0 or Graphpad Prism 6. One‐way ANOVA, univariate Kaplan‐Meier method followed by log‐rank test and Chi‐squared test were used to calculate the difference between groups. *P* < 0.05 was considered statistically significant.

## Results

### MiR‐374b expression reduced in NSCLC

**Figure 1:**
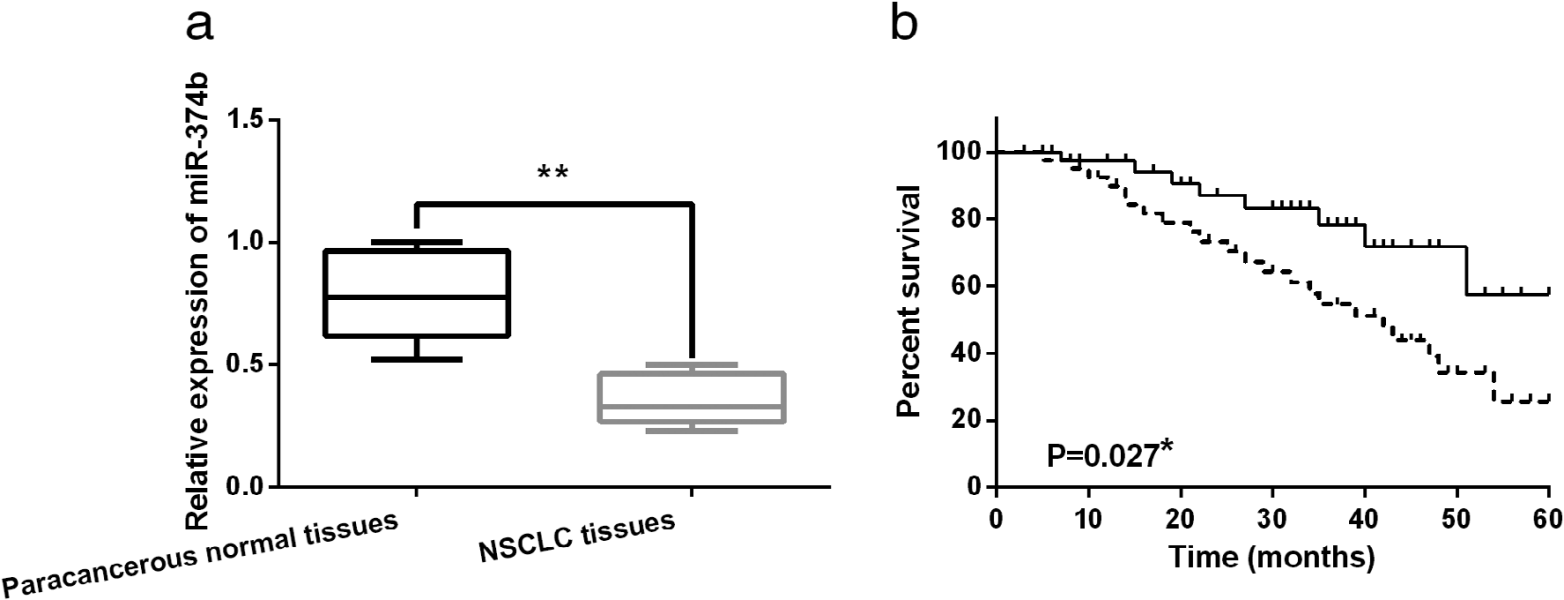
MiR‐374b expression was reduced in non‐small cell lung cancer (NSCLC). (**a**) MiR‐374b expressions in NSCLC tissues and paracancerous normal tissues. (**b**) Unfavorable prognosis in NSCLC patients with low miR‐374b expression. *P < 0.05, **P < 0.01.

First, the abnormal expression of, miR‐374b was observed in NSCLC. Compared to normal tissues, miR‐374b expression was decreased in NSCLC tissues (Fig 1a). In addition, it was found that the low expression of miR‐374b was correlated with the aggressive behavior of NSCLC patients including lymph nodes metastasis or tumor stage (Table 2). Importantly, low expression of miR‐374b was associated with poor prognosis in NSCLC patients (Fig 1b). Combining these results, we suspected that miR‐374b may regulate the tumorigenesis of NSCLC.

**Table 2:** Relationship between miR‐374b expression and clinicopathological characteristics in NSCLC patients

Characteristics	Cases	miR‐374b		*P*‐value
		High	Low	
Age (years)				0.08
≥ 60	44	14	30	
<60	38	17	21	
Gender				0.11
Male	43	14	29	
Female	39	17	22	
Tumor size (mm)				0.12
≤3	52	22	30	
>3	30	9	21	
Lymph nodes metastasis				0.012*
Yes	17	7	10	
No	65	24	41	
Tumor stage				0.023*
I–II	60	24	36	
III–IV	22	7	15	

Statistical analyses were performed by the χ^2^ test

* *P*<0.05 was considered statistically significant.

### Overexpression of miR‐374b inhibits NSCLC cell viability and metastasis

**Figure 2:**
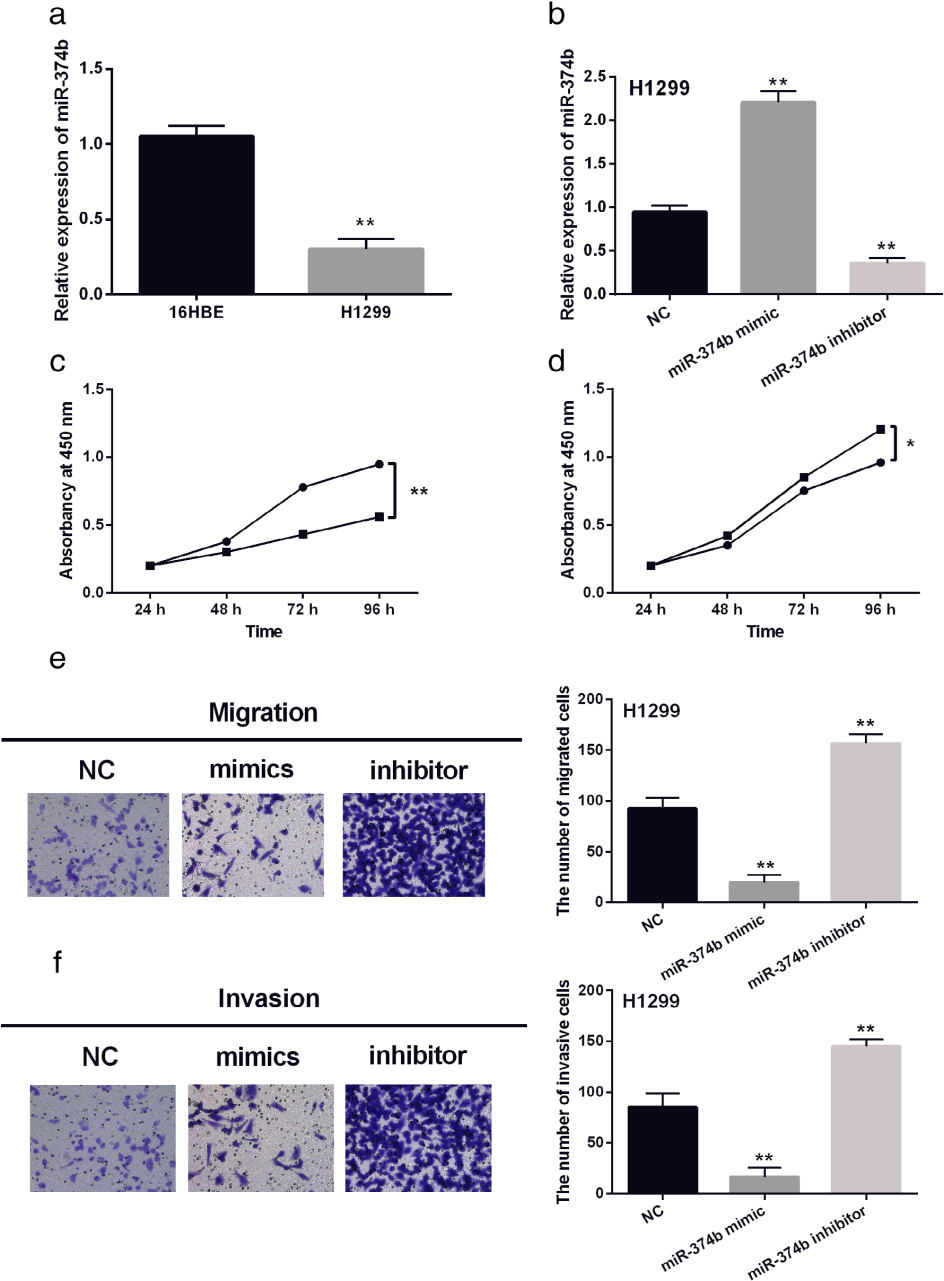
MiR‐374b overexpression restrained non‐small cell lung cancer (NSCLC) cell viability and metastasis. (**a**) MiR‐374b expression in H1299 and 16HBE cell lines (**b**) MiR‐374b mimics or inhibitor regulated its expression in H1299 cells (**c**, **d**, **e**, **f**) Cell proliferation, migration and invasion in H1299 cells with miR‐374b mimics or inhibitor. Untreated H1299 cells were used as the negative control (NC). *P < 0.05, **P < 0.01.

Next, the expression of miR‐374b was examined in H1299 and 16HBE cell lines. Compared to 16HBE cells, miR‐374b was downregulated in H1299 NSCLC cells (Fig 2a). The transfection efficiency of miR‐374b mimics or inhibitor in H1299 cells was then detected by RT‐qPCR (Fig 2b). Functionally, upregulation of miR‐374b inhibited the proliferation of H1299 cells (Fig 2c). Downregulation of miR‐374b promoted cell proliferation in H1299 cells (Fig 2d). Similarly, overexpression of miR‐374b inhibited cell migration, while downregulation of miR‐374b promoted cell migration in H1299 cells (Fig 2e). Meanwhile, cell invasion showed the same results as cell migration in H1299 cells (Fig 2f). Therefore, overexpression of miR‐374b inhibits cell viability and metastasis in NSCLC.

### MiR‐374b blocks EMT and promotes p53 expression in NSCLC

**Figure 3:**
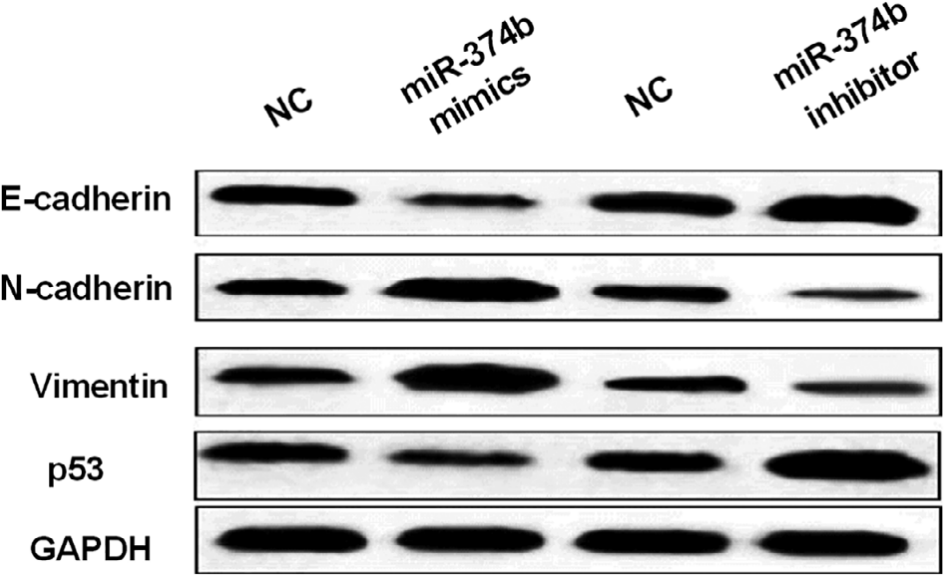
MiR‐374b blocked EMT and promoted p53 in non‐small cell lung cancer (NSCLC). MiR‐374b regulated Vimentin, N‐cadherin, E‐cadherin, and p53 expressions in H1299 cells. Untreated H1299 cells were used as the negative control (NC).

Whether miR‐374b regulates EMT and tumor suppressor p53 in NSCLC was then investigated. The expressions of N‐cadherin and Vimentin was suppressed by miR‐374b upregulation, but was promoted by miR‐374b inhibitor in H1299 cells (Fig 3). In addition, miR‐374b overexpression promoted E‐cadherin expression, while knockdown of miR‐374b suppressed E‐cadherin expression in H1299 cells (Fig 3). In addition, it is well known that p53 acts as a tumor suppressor in human cancer. Therefore, we investigated how miR‐374b regulates p53 expression in H1299 cells. We found that miR‐374b overexpression promoted p53 expression, but miR‐374b knockdown suppressed p53 expression (Fig 3). miR‐374b may therefore inhibit NSCLC progression by blocking EMT and upregulating p53.

### ITGB1 is a direct target for miR‐374b

**Figure 4:**
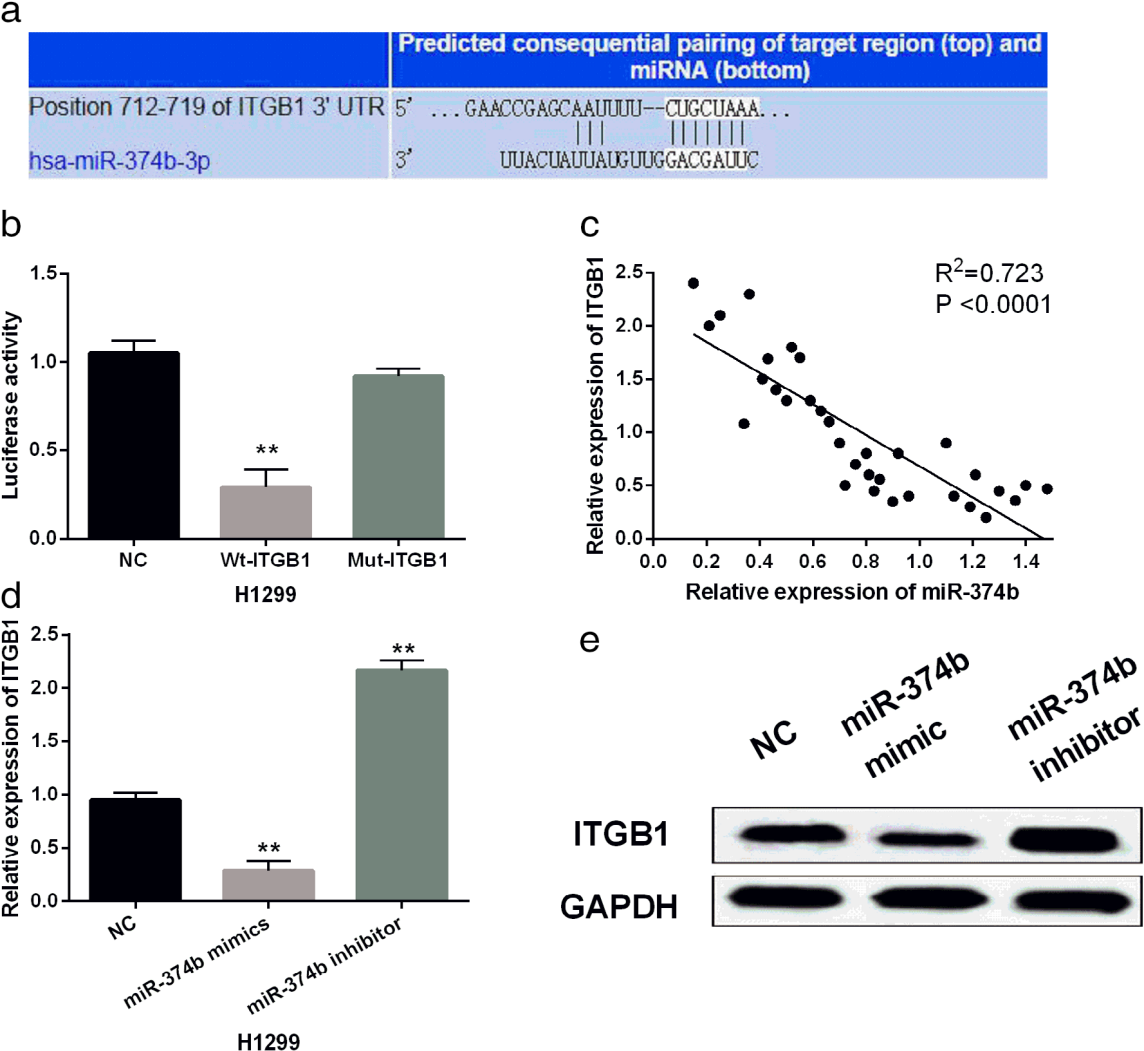
MiR‐374b directly targets ITGB1. (**a**) The binding sites between miR‐374b and ITGB1. (**b**) Luciferase reporter assay (**c**) MiR‐374b negatively regulated ITGB1 expression in NSCLC tissues. (**d**, **e**) MiR‐374b regulated ITGB1 expression in H1299 cells. Untreated H1299 cells were used as the negative control (NC). **P < 0.01.

The TargetScan (http://www.targetscan.org/) database shows that miR‐374b has a binding site to ITGB1 (Fig 4a). Luciferase reporter assay showed that miR‐374b mimics significantly inhibited the luciferase activity of Wt‐ITGB1 (Fig 4b), but had little effect on that of mut‐ITGB1, indicating that miR‐374b directly targets ITGB1. As shown in Fig 4c, miR‐374b was found to be negatively correlated with ITGB1 expression in NSCLC tissues. The expression of ITGB1 in transfected H1299 cells was evaluated. Overexpression of miR‐374b inhibited ITGB1 expression, while downregulation of miR‐374b promoted ITGB1 expression in H1299 cells (Fig 4d,e). Collectively, miR‐374b directly targets ITGB1 and negatively regulates the expression of ITGB1 in NSCLC.

### MiR‐374b inhibits the progression of NSCLC through inhibition of ITGB1

**Figure 5:**
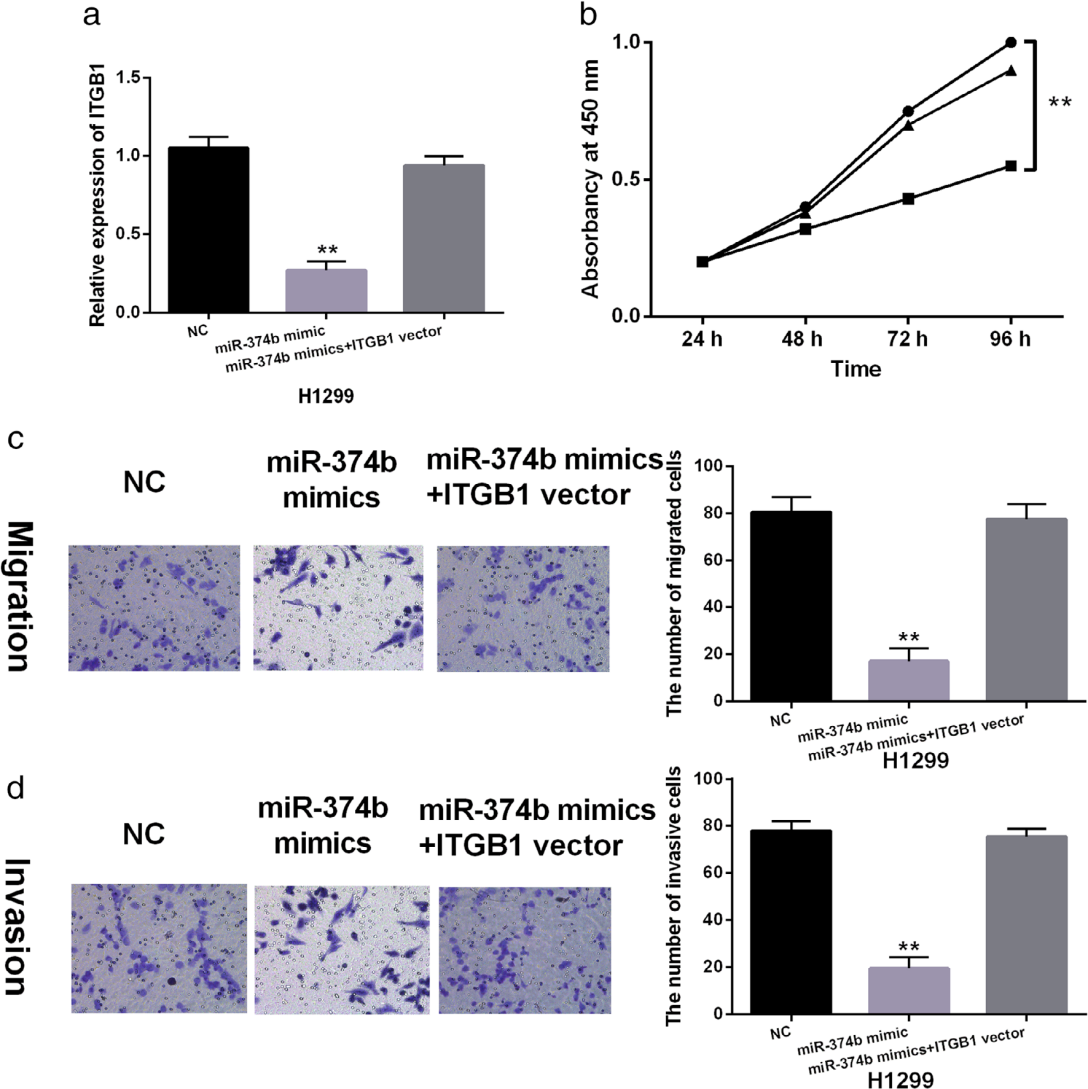
MiR‐374b blocked non‐small cell lung cancer (NSCLC) progression through inhibition of ITGB1. (**a**) ITGB1 expression in H1299 cells with miR‐374b mimics and ITGB1 vector. (**b**, **c**, **d**) Cell proliferation, migration and invasion were detected in H1299 cells harboring miR‐374b mimics and ITGB1 vector. Untreated H1299 cells were used as the negative control (NC). **P < 0.01. 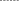 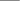

To explore the interaction between miR‐374b and ITGB1 in NSCLC, a rescue experiment was performed in H1299 cells. We found that the decreased expression of ITGB1 induced by miR‐374b mimics was restored by ITGB1 vector in H1299 cells (Fig 5a). Functionally, upregulation of ITGB1 weakened the inhibitory effect of miR‐374b on cell proliferation in H1299 cells (Fig 5b). Meanwhile, the inhibitory effect of miR‐374b on cell invasion and migration was attenuated by ITGB1 overexpression in H1299 cells (Fig 5c,d). Overall, miR‐374b inhibits the tumorigenesis of NSCLC by downregulating ITGB1.

## Discussion

As antitumor genes or oncogenes, many miRNAs have been reported to regulate various biological processes of NSCLC cells, such as miR‐1269a and miR‐212.^18^, ^19^ In the current study, miR‐374b expression was reduced in NSCLC tissues and downregulation of miR‐374b was associated with poor clinical outcomes and prognosis in NSCLC patients. Importantly, miR‐374b was found to inhibit cell viability and metastasis in NSCLC. In addition, miR‐374b inhibited EMT and promoted p53 expression in NSCLC. Further, miR‐374b directly targets ITGB1. Upregulation of ITGB1 attenuated the antitumor effect of miR‐374b in NSCLC. Briefly, miR‐374b inhibited the tumorigenesis of NSCLC by regulating the expression of ITGB1 and p53.

Previous studies have reported the downregulation of miR‐374b in pancreatic cancer, cervical cancer and colorectal cancer,^20^, ^21^, ^22^ which is consistent with our results. Functionally, miR‐374b restrained the progression of liver cancer by inhibiting PD‐1.^23^ Moreover, miR‐374b reduced the proliferation and invasion of colon cancer cells.^24^ It has also been reported that the miR‐374b/FOXP1 axis suppressed EMT and cell migration in ovarian cancer.^25^ Similar results were also detected in NSCLC. In addition, Gong *et al*. reported that p53 can enhance the expression of miR‐374b in colorectal carcinoma.^10^ Here, it was found that miR‐374b overexpression promoted p53 expression in NSCLC. At the same time, many studies have shown that miR‐374b exerts its effect by inhibiting the expression of target genes, such as AKT1 and PTEN.^26^, ^27^ In this study, it was found that miR‐374b directly targets ITGB1.

Some miRNAs have been shown to target ITGB1, such as miR‐134 and miR‐29.^28^, ^29^ In addition, upregulation of ITGB1 has been detected in pancreatic ductal adenocarcinoma and nasopharyngeal carcinoma.^30^, ^31^ Here, we also found that ITGB1 expression was upregulated and negatively regulated by miR‐374b in NSCLC. Similar to our results, miR‐124 also suppressed OSCC cell migration and invasion by targeting ITGB1.^32^ miR‐223 inhibited cell invasion and migration in prostate cancer by downregulating ITGB1.^33^ Meanwhile, miR‐29c was found to inhibit the growth of pancreatic cancer cells by inhibiting ITGB1.^34^ In our study, miR‐374b inhibited cell viability, migration and invasion in NSCLC by downregulating ITGB1. In addition, high expression of ITGB1 and low expression of p53 has been found to act as adverse prognostic factors in NSCLC.^35^ P53‐induced miR‐30e‐5p inhibits cell metastasis in colorectal cancer by targeting ITGB1.^36^ All these findings support our conclusion that miR‐374b inhibits the progression of NSCLC by downregulating ITGB1 and promoting p53 expression.

In conclusion, miR‐374b expression was reduced in NSCLC. Functionally, miR‐374b overexpression restrained the viability and metastasis of NSCLC cells. Overexpression of miR‐374b inhibited EMT and promoted p53 expression in NSCLC. Importantly, miR‐374b inhibited the progression of NSCLC by targeting ITGB1. This study may help us understand the tumor formation mechanism of NSCLC.

## Disclosure

The authors declare that they have no competing interests.
